# Appendicitis, Typhitis and Perityphlitic Abscess*Read before the Wisconsin State Medical Society, May 5th, 1892.

**Published:** 1892-07

**Authors:** A. H. Levings

**Affiliations:** Milwaukee, Wis.


### Southern Medical Record.

A MONTHLY JOURNAL OF MEDICINE AND SURGERY.

VOL. XXII. ATLANTA, GA., JULY, 1892. No. 7

### Original Articles.

### APPENDICITIS' TYPHLITIS AND PERITYPHLITIC ABSCESS.*

BY A. H. LEVINGS, M. D., MILWAUKEE, WIS.

To the surgeons of France must be awarded the credit of having first called attention to the relationship between abscesses in the right iliac region and diseased conditions of the cæcum or appendix vermiformis.

Dupaytren, in 1820, directed attention to the diseased conditions of the cæcum as a cause of abscess in the right iliac fossa. Loyer and Villermay, in 1824, to perforation of the vermiform appendix as a cause of acute general peritonitis. Shortly afterwards, Puchelt, of Germany, gave to the inflammatory conditions in the right iliac fossa the name perityphlitis, which strictly speaking, means an inflammation of the peritoneum covering the cæcum, but by general consent it has come to represent a localized peritonitis originating either in the cæcum or appendix, and implicating adjacent layers of peritoneum, but confined to the right iliac fossa. Should localized suppuration be established in this inflamed region, a perityphlitic abscess is produced.

Oppolzer, in 1863, brought forward the term perityphlitis,

*Read before the Wisconsin State Medical Society, May 5th, 1892.

indicating an inflammation of the connective tissue behind the cæcum. A faithful history of the anatomy, pathology and treatment of suppurative processes in the right iliac fossa could, however, not be written without making mention of many eminent American and English surgeons and physicians, and among the most prominent should be the name of our own countryman, the late Prof. Willard Parker, who, in 1843, was the first to operate in a surgical sense for a perityphlitic abscess. Hancock, of London, operated in 1848, and advocated the operation in an article in the London Medical Gazette, and he is by many given the credit of having originated the operation. It was, however, generally discredited until Willard Parker, in 1867, reported four cases treated by incision, of which number three recovered. Probably no one man has done more to place these inflammatory processes in their true anatomical and pathological light than Frederick Treves, in his Hunterian lectures on the "Anatomy of the Intestinal Canal and Peritoneum in Man," as well also as in his subsequent writings.

ANATOMY OF THE CÆCUM AND APPENDIX REFLECTION OF THE PERITONEUM.

The cæcum, when in its normal position, is in the right iliac region, and is that portion of the large intestine placed below the entrance of the ilium at the ilio-cæcal valve. It rests upon and its tip just projects over the internal border of the psoas muscle. Its lower extremity will just reach a line drawn from the middle of Poupart's ligament to the umbilicus at the junction of its middle and lower thirds.

The cæcum, is as a rule, about three inches in breadth and two and one-fourth inches in length. The ilium enters it at right angles from the left.

Text books on anatomy have taught that the peritoneum was reflected over the anterior portion of the cæcum, leaving its posterior portion uncovered, but connected to the iliac region by means of cellular tissue.

Bardeleben, in 1849, from an examination of one hundred and sixty cadavers, asserted that the cæcum was completely surrounded by peritoneum; also the celebrated anatomist

Laschke, in 1861, declared that in the fœtus the peritoneum surrounds entirely the cæcum and appendix, and that the same condition prevails as a rule in adult life.

The assertions of these two distinguished anatomists, however, attracted little attention, and it was not until Frederick Treves, in 1885, from an examination of one hundred bodies, most positively asserted that the cæcum is entirely covered by peritoneum; that this view became the generally accepted one, both by anatomists and surgeons.

In the eleventh edition of Gray's Anatomy, published in in 1887, the reflections of the peritoneum as given by Treves are adopted, with the statement that they are unquestionably correct. This being true, the term paratyphlitis is evidently anatomically incorrect, as all inflammatory processes arising from either appendix or cæcum must primarily be intra peritoneal.

The appendix vermiformis is attached to the lower and back part of the cæcum. It is a long, narrow, worm-like process, usually coiled upon itself, measuring from three to six inches in length, and about the size of a lead pencil. It terminates in a blunt point and its canal communicates with the cæcum by a small orifice, which is often guarded by a fold of mucous membrane called Garlach's valve. The coats of the cæcum and appendix are the same as those of the large intestine. The mucous membrane, however, has a greater number of solitary glands, and in the appendix there are many Peyer's patches. The appendix is entirely surrounded by peritoneum, which generally forms for it a mesentery. Its direction is usually upwards and inwards, behind the internal border of the cæcum or lying on the mesentery of the ilium.

The usual position of the cæcum as stated, is lying on the psoas muscle, with its apex just projecting over its internal border (Treves), although this may be said to be its usual position, its actual position as well as its mobility in any given case depends on the height of the reflection of the peritoneum from the ascending colon, and on the existence and length of the ascending meso-colon.

In a case which I recently examined post-mortem, the cæcum was found resting on the bladder, its apex not more than one inch from the symphysis pubis. The appendix was hanging free in the pelvis. In this case the cæcum could be made to touch almost any part of the abdominal cavity. Treves states that in eleven of the one hundred bodies he examined, the cæcum could be made to touch the under surface of the liver and any part of the left side of the abdomen, and in seven instances it could be drawn down the thigh as far as the great trochanter. The reflection of the peritoneum from the posterior surface of the cæcum on to the posterior abdominal parietes corresponds to the commencement of the ascending meso-colon.

In the post-mortem examinations that I have made, there have been found three principal folds formed in the reflection of the peritoneum, which are shown in their respective cases on raising the cæcum. In the first and most frequent, a fold of peritoneum is seen reflected from the ascending meso-colon along the line of the psoas muscle, forming the external boundary of a distinct fossa, which is bounded internally by the reflected mesentery from the terminal portion of the ilium. In this fossa would be fouud the appendix. The mobility of the appendix will depend entirely on the length of its mesentery.

In the second class of cases, the reflection of the peritoneum would follow two distinct lines ; one, the internal fold would be as before, along the border of the psoas muscle, and the other, the external, would be from the external border of the ascending colon outwards towards the anterior superior spine of the ilium. In this very distinct fossa directly underneath the cæcum would be found the appendix, probably entirely hidden from view unless the cæcum was raised.

In the third class of cases, the reflections of the peritoneum from the meso-colon would be in a fold along the external border of the psoas muscle, and the appendix would be found external to this fold, or along the outer border of the cæcum.

In six cases recently operated on by McBurney, he reported finding the appendix in two of them in this position.

The appendix may, however, be found in the pelvis in contact with the sigmoid flexure, rectum, uterus or bladder. The fossæ described as produced by the reflections of the peritoneum from the commencement of the ascending colon, are called iliac fossæ and are of great surgical interest, as small collections of pus or the appendix may be circumscribed or bound down by adhesions in one of them and thus hidden from view.

In accordance with former anatomical teaching, that the posterior portion of the cæcum and a part of the appendix were not covered by peritoneum, it was also naturally taught that the perityphlitic abscess was extra-peritoneal. Now, on the contrary, it is known that these abscesses are primarily always intra-peritoneal and only become extra-peritoneal, if they do so at all, by the extension of the suppurative process.

ETIOLOGY.

The frequency with which inflammatory action is established in the appendix and cæcum must very largely depend on their anatomical arrangement, the small intestine opening into the large would in consequence of the disparity in size of the two segments, tend very materially to produce stagnation of fæcal matter at this point. Added to this is the fact of the cæcum and appendix being placed below the entrance of the ilium, below the natural fæcal current. They are, indeed, pockets most favorably placed for the lodgment and retention of fermenting fæcal matter, foreign bodies and pathogenic germs. This lodgment and retention of fæcal material is productive of mechanical irritation, catarrhal inflammation, superficial tissue, necrosis, septic inflammation and ulceration which leads or may lead to gangrene or perforation.

Treves says that the appendix is obsolete and out of date, and it is safe to predict that in the intestine of the man of the future there will be no such process found hanging from the cæcum. Sex and age play a very important part. Three-fourths of all cases occur in males. In 1030 cases collected by Matterstock, 63 per cent. occurred between the ages of eleven and thirty.

The fold of mucous membrane placed at the entrance of the appendix contracting its orifice, is most prominent during adult life, at the age that appendicitis is most frequent.

Gerlach, whose name this valve bears, believes that it, by constricting the orifice of the canal, hinders the expulsion of materials from the appendix, and thereby favors inflammation and perforation.

Constipation and intestinal catarrh in producing fæcal concretion and retention of fæcal material in these parts, unquestionably plays a most important part in typhlitis and appendicitis. Tubercular, typhoid and dysenteric ulceration may lead to perforation of the cæcum or appendix. Matterstock has collected one hundred and forty-six cases of perforation of the appendix, in sixty-three of which concretions were found, and in nine cases foreign bodies. Fitz, in one hundred and forty-two cases in children and adults, found fæcal concretions in forty-seven per cent., and foreign bodies in twelve per cent. The concretions may be composed entirely of fæcal matter and be soft, or they may be in part composed of phosphate or carbonate of lime and hard, or they may have one of the various fruit seeds for a nucleus. Among the foreign bodies producing perforation, the seeds of fruits are the most frequent. Cæcal stones containing calcareous matter and very hard are mentioned by Albers and others as having produced perforation of the cæcum.

### TRAUMATISM.

In one hundred and one cases out of two hundred and nine, external violence immediately preceded the attack (Fitz). The lifting of heavy weights, a fall, violent exercise and overeating are frequently supposed to be causes.

### PATHOLOGICAL ANATOMY.

The primary changes in the appendix or cæcum from fæcal concretions or foreign bodies, are first, mechanical irritation leading to catarrh. The process may stop here, or the cause being continued, there will be superficial necrosis of the epithelial layers, affording an excellent opportunity for the invasion of pathogenic germs. The extent and rapidity of the process will depend on the number and virulence of these germs, or their ptomaines. In the worst cases, gangrene of the entire appendix without limiting adhesion, and with the symptoms of severe sepsis may occur, or a large perforation may be produced, followed by acute general peritonitis, limiting adhesion not having formed. In less acute cases, limiting adhesions are formed from a fibrinous exudate surrounding the appendix, or a perforating ulcer of the cæcum, and shutting off the general peritoneal cavity. In this way a circumscribed perityphlitic abscess is produced. In cases where the germs are less virulent or fewer in number, and these are probably by far the most numerous cases, the exudate on the adjacent surfaces of the peritoneum remains plastic without the formation of pus. This exudate may bind down the appendix, producing twists or kinks in its lumen. Often in consequence of interstitial inflammation, its canal is much narrower, or even obliterated in whole or in part—as a result of this partial obliteration, retention cysts are often produced.

In relapsing appendicitis there is usually some stenosis of the canal, associated with a catarrhal inflammation and a plastic, localized peritonitis. The tendency to the formation of pus in these cases is very slight, although recently McBurney has reported one such case, and Abbe two. Nature's methods of cure in perityphlitic abscesses are very various. They frequently discharge into the cæcum, or may discharge into the small intestine or rectum, with good results. The bladder is occasionally perforated, a fæcal cystitis with fatal result usually occurs. They often discharge through the abdominal wall, and in children at the umbilicus. An abscess placed on the outside of the cæcum may extend upwards to and behind the liver, producing a sub-diaphragmatic abscess. When in this situation it may perforate the pleura.

These abscesses are primarily always intra-peritoneal, but by an extention of the suppurative process, they may penetrate the posterior parietal peritoneum and become extra-peritoneal or perityphlitic. In this situation behind the cæcum they can extend upwards to the kidney, producing a perinephritic abscess, or following the psoas muscle point beneath and below Poupart's ligament. The pus may find an exit through the obturator foramen forming an abscess under the gluteal muscles or on the thigh, or enter the hip-joint, or follow the rectum and become a peri-rectal abscess. Powell reports a case where the abscess communicated with the internal iliac artery, and Bryant a case of communication with the deep circumflex artery. The two most fatal extensions of perityphlitic abscesses are, first, perforation into the general peritoneal cavity producing acute diffuse peritonitis. This occurred in six of thirty cases reported by Wythe, and in eight out of sixty-seven cases reported by Bull. Second, an extension of the inflammation to the ilio-cæcal vein, with the result of producing a septic phlebitis of this and the portal vein and abscesses of the liver. This is not a very uncommon and a most fatal result.

SYMPTOMS.

Typhlitis stercoralis is usually a chronic affection with a history of constipation, alternating perhaps with occasional attacks of diarrhœa, and attended with colicky pains in the ileo-cæcal region. The general health is somewhat disturbed, occasionally there are acute exacerbations, the pain is increased, there may be fever and vomiting. The patient is disinclined to exertion, as it increases the pain; the appetite is poor. The most important signs are gained from a physical examination. The bowels are usually distended with gas. In the cæcal region at the commencement of the attack, will be found a tumor slightly sensitive on pressure; large, extending up towards the liver and of a doughy feel. Typhlitis stercoralis may advance to perforation of the bowel, attended with the acute symptoms of a perityphlitis, but this result is very unusual. Its symptoms are usually mild and its course chronic. It should be differentiated from acute appendicitis as well as from acute perforating ulcers of the cæcum. It is now well established that the great majority of inflammatory and suppurative processes in the right iliac fossa have their origin in the appendix.

Appendicitis has occasionally a prodromal stage. Its symptoms are colicky pains, with irregularity of the bowels, pains after eating and a variable appetite. An acute attack is ushered in by sudden severe agonizing pain starting from the cæcal region. It may radiate through different parts of the abdomen, or the pain may be at first general or more intense at the umbilicus. The pain may be very severe during the first day or night and then gradually subside, leaving only sensitiveness on pressure over the cæcum; or the pain may come on gradually, and constantly increase during the course of the disease. There is usually nausea and vomiting, at first the contents of the stomach are ejected, afterwards bile, but never stercoraceous material. Disorder of the stomach is more constant and severe in children than in adults. There is loss of appetite, the bowels are usually obstinately constipated, although constipation may alternate with diarrhœa. The temperature generally ranges from 100 degs. F. to 102. The pulse is accelerated, ranging from 100 to 120. In children, convulsions and delirium may be present.

The physical signs are very important in making a diagnosis. At first there is slight tympanic distention of the bowels, without dullness. The abdominal muscles on the right side are tense. There is increased sensitiveness in the cæcal region, and firm pressure increases the pain. McBurney has pointed out that on a line running from the anterior superior spine of the ilium to the umbilicus, and two inches internal to the spine, finger tip pressure will always elicit pain. This point varies somewhat with the location of the appendix, but nevertheless the sign is a very valuable one.

A tympanitic dullness is usually observed over the cæcum after the third day. In thirty-seven cases of typhlitis, appendicitis and perityphlitis, Fitz records dullness as occuring on the first day in two cases, on the second in two, on the third in eight, on the fourth in nine, on the fifth in three, on the sixth in two, on the seventh in two, on the eighth in five, on the ninth in one and on the tenth day in three cases.

As regards the first appearance of a tumor, the same author found that palpation showed the presence of a tumor in ninety-two cases as follows: on the first day in five cases, on the second in nine, on the third in twelve, on the fourth in twelve, on the fifth in seven, on the sixth in eleven, on the seventh in eight, on the eighth in eight, on the ninth in eleven and on the tenth day in eleven cases. Fluctuation not occurring until after the second week, is of no special diagnostic value.

DIAGNOSIS AND DIFFERENTIAL DIAGNOSIS.

The diagnosis of perityphlitis is as a rule comparatively easy, but that it may be at times extremely difficult and sometimes well nigh impossible even in the most skilled hands, is sufficiently attested by the numerous autopsies or operations performed, where there was a failure to make a correct diagnosis. This may in a measure be accounted for by its variable symptoms, as well as from what may be termed the unnatural location of the cæcum and appendix, due largely to the mobility of the cæcum. However, if the cardinal symptoms are kept well in mind, one will not be long in coming to a correct diagnosis in any given case.

The most important and most constant symptoms are sudden severe pains in the cæcal region, attended with fever and vomiting, tension of the muscles and increased sensitiveness on pressure over the cæcum, and the finger tip pressure pointed out by McBurney, with constipation, and often on the third day, a tumor.

The diseases most likely to be mistaken for perityphlitis are peritonitis, invagination, intestinal obstruction, psoas abscess, perinephritic abscess and hip-joint disease.

Peritoneal inflammation originating in other parts of the abdominal cavity, then the right iliac region, is as a rule, not of appendicular origin; while peritoneal inflammations, having their starting point in the cæcal region, especially in man, are almost invariably due to disease of the appendix. In invagination there will usually be found a movable tumor in the left side, without fever for the first days, and attended, especially in children, with tenesmus and bloody discharges from the bowels.

In strangulation, vomiting is most obstinate and becomes stercoraceous from the third to the fifth day. Constipation is absolute beyond the contents of the large bowel. There is no fever until commencing peritonitis. The disease affects the system more profoundly than does a case of perityphlitis. Psoas abscess may compare in position to a perityphlitic abscess, but its course is chronic, of slow development, attended with disease of the spine; fluctuation can usually be made out, the abscess is sub-peritoneal, following the course of the psoas muscle and pointing below Poupart's ligament.

In peri-nephritic abscess, the disease is located in the groin and is usually associated with pus in the urine.

HIP-JOINT DISEASE.

Under ordinary circumstances, it would seem inexcusable to mistake these diseases, still Gibney reports four cases of perityphlitis being mistaken for hip-joint disease. In acute cases, all that they will have in common will be fever and flexion of the thigh, with pain on extension. In cases of hip-joint disease that has gone to perforation of the acetabulum and the formation of an iliac abscess, the disease will have been of weeks or months duration. The tumor will be lower down nearer Poupart's ligament, will often fluctuate and being sub-peritoneal, will tend to point below Poupart's ligament. The prognosis in any given case of appendicitis will largely depend on the acuteness of the process, the size of the perforation, if one takes place, and as to whether suppuration is established or not. In very acute cases with gangrene of the appendix, the result will probably be always fatal without timely interference by operation. In perforation without limiting adhesions producing acute general peritonitis, the result has been almost invariably fatal. Although Dr. Abbe has recently reported that he has cured two cases of acute general peritonitis by making an opening in each groin and keeping up constant irrigation.

According to Fitz's tables, thirty-two per cent. undergo resolution without the formation of pus, and in eighteen per cent. with the formation of pus, spontaneous evacuation takes place, leaving fifty per cent. dependent on operation. There can be no doubt but what very many cases of appendicitis are recovered from, without the condition ever having been diagnosed. In many cases the post-mortem table reveals evidences of previously existing appendicitis from which the patients had recovered without having been aware of the disease from which they suffered. Toft, in 300 autopsies, reports that 36 per cent. revealed evidence of disease of the appendix. Also Dr. Ludwig Hexton (Bridge) in 280 autopsies found in 15 per cent evidences of previous peri-appendicitis from which the patient had recovered.

TREATMENT—MEDICAL.

There is still some diversity of opinion in regard to the medical treatment of perityphlitic processes. On one point all are agreed that in stercoral typhlitis, the bowels should be thoroughly evacuated either by purgatives or enemata, or by both.

In that form of perityphlitis caused by inflammation or perforation of the appendix, the majority of authors discountenance the use of both purgatives and enemata, holding that by their use the recently formed limiting adhesion will be rendered liable to rupture in consequence of the increased peristalsis. These physicians give opium in sufficient quantity to allay pain, produce sleep and hinder the peristaltic action of the bowels, hoping thereby to favor the formation of limiting adhesions. Among the physicians recommending the above line of treatment may be mentioned Fitz, Matterstock, Wythe, Volz, Stokes and Graves, Loomis, Whittaker, Flint, Strumbell and Jacobi.

Meigs and Pepper give mild salines, with opium for rest. Silberman gives in children hydrate of chloral instead of opium, Monti uses irrigation of the bowels with purgatives.

DEPLETION.

The German physicians as a rule, such as Nothnagel, Strumpell and Matterstock use leeches or wet cups, followed by the ice bag, Bull uses leeches, Balzer and Gibney recommend blisters.

Warm poultices or hot compresses are much used in this country. Their use, although in a measure allaying pain, is extremely questionable, unless for the purpose of aiding or hastening suppuration. The diet should be liquid and absolute rest be maintained. During convalescence also, the greatest circumspection should be exercised both as regards diet and exercise.

Nearly all cases are primarily medical. Just where and when the distinction should be drawn between medical and surgical cases is still a matter of dispute.

Stercoral typhlitis will undoubtedly always remain medical, Cases of plastic appendicitis without the formation of pus and without severe recurrences will remain medical. The recurrent cases of catarrhal appendicitis will be medical until it is shown that the recurrences are sufficiently often and severe to affect the general health and to prove disabling, then they will become surgical. Cases of acute gangrene of the appendix should always be surgical, as well as cases of acute perforation without limiting adhesions, although there is still some contension over the suppurative cases, nevertheless the practice of treating all cases in which pus has formed as surgical, is fast becoming the recognized rule.

The cases demanding operation may be divided into three classes. In the first will be the extra-acute cases. The operation is done for the removal of either a gangrenous or perforated appendix. The symptoms are alarming and threaten either general sepsis or general peritonitis. The operation is done on the second or third day, and is preventive in its scope, little or no pus will be found, and often by abdominal palpation, no tumor will be made out. The incision must be a long one, from four to five inches, and placed over the most sensitive point determined by finger tip pressure, this will be over the cæcum. The peritoneal cavity is deliberately opened and search is made for the appendix. It will usually be found inclosed in a quantity of recent exudate forming a small tumor, in which there may be a small amount of pus. This tumor should be carefully surrounded with sponges or sponge cloths before being broken into, so as to catch and remove every particle of pus, thereby preventing infection of the general peritoneum. After being thus surrounded, it is broken into, the pus removed, the adhesions binding down the appendix broken up, the appendix ligated near its junction with the cæcum and cut away. McBurney practices disinfecting the cæcal extremity by means of the cautery. The small cavity is then carefully dried with sponges and packed with iodoform gauze, the strip being brought out of the wound, which is partially closed, and an antiseptic dressing applied. The dressings are changed every two to four days and the healing is complete in about a month.

At the present time this operation is confined to the extraacute cases, where it would be extremely hazardous to wait for limiting adhesion. It is distinctly an American operation, brought forward and perfected by the genius and skill of American surgeons. The results even in most desperate cases have been thus far most gratifying.

In the second class of cases, the symptoms, though acute and pronounced, are more indicative of a localized peritonitis. In these cases the operation is usually deferred until from the fourth to the eighth day, after a tumor and limiting adhesion have formed. The incision, some two inches long, is made directly over the most prominent part of the tumor, and on reaching the tranversalis fascia,it has been quite the rule to use the hypodermic needle with the view of locating the pus or ascertaining if any existed. The procedure is now not very generally in use, for the reason that pus may be present even in considerable quantity and the needle fail to show it. But a few days since I assisted at an operation for perityphlitis where the needle was used, but failed to show pus, and on incising the peritoneum a liberal quantity of pus was discharged. Sands was the first to draw attention to the possible dangers of the needle, in that it might perforate coils of adherent intestine before entering the abscess cavity, and on being withdrawn, infect the peritoneum. I recently operated on a case in which adherent coils of intestine were encountered directly beneath the peritoneum, over the most prominent part of the tumor. On separating these and some recent adhesion to the cæcum, at least one-half of a pint of pus was liberated from an abscess placed directly underneath the cæcum. In this case it would have been anatomically impossible to have reached the pus with a needle without first perforating at least two or more coats of intestine. It has also been recommended after locating pus with the needle to leave it in sight as a guide, and cut down on the pus along the needle. Should the needle have perforated one or more coils of intestine, the result would not be gratifying to the surgeon.

If the peritoneum is incised and pus is not found directly beneath it, the wound must be enlarged, the adherent coils of intestine carefully separated with the fingers towards the center of the tumor until pus is reached. If the appendix is found lying loose in the abscess cavity, it should be ligated and removed. If it is adherent, it should not be disturbed; only the gentlest manipulation in the abscess cavity is permissible, as several cases are on record of rupture from manipulation into the general peritoneal cavity and death.

The abscess cavity is usually irrigated with some mild antiseptic solution, a drainage tube introduced and a large antiseptic dressing applied.

RECURRENT APPENDICITIS.

The medical treatment is confined to regulating the bowels, the diet, and the exercise and giving opium for pains.

Treves was the originator of the operation for removal of the appendix between the attacks in cases of recurrent appendicitis. In suitable cases the operation is generally recommended, although Frederick S. Dennis, in an elaborate article in the *Medical News*, opposes it, and contends that if an operation is to be performed it should be during the attack. The majority of operators, however, followed Treves. The indications for the operation are severe recurrent attacks producing invalidism. The operation is performed in the usual way with a rather long incision over and parallel with the cæcum. The appendix is separated from its adhesions, tied and removed, or after being incised its end is closed with a row of Lambert's sutures. The wound is closed without drainage. The mortality has been almost *nil*, and the results most gratifying.

